# Samaritan Free Hospital for Women

**Published:** 1915-10-09

**Authors:** 


### THE DEPARTMENT OF MODERN NURSING.*

### Samaritan Free Hospital for Women.

The training given to probationers at the Samaritan Hospital is described by the matron as "preliminary," perhaps because it is confined to the treatment of women only, but, with this limitation, the surgical and gynæcological training is very extensive and thorough, the number of major operations being unusually large.

Probationers are received between the ages of twenty and thirty-five—more often at twenty—thus filling up their time till they are old enough for a general hospital, a goal to which almost all the Samaritan nurses attain. About ten are received in a year, and the staff consists of eighteen probationers, three staff-nurses, a theatre-sister, night-sister, and two ward-sisters, a total of twenty-six nurses for sixty-five beds. The matron has, so far, had no need to advertise for probationers, as candidates often come by the recommendation of the matrons of general hospitals, who find them too young to join their staff. A certain number are sent on for six months only by the City of London Infirmary in order that the training of their nurses may be completed by this period of surgical work. Others again are nurses of six months' experience in maternity hospitals, who have passed the examination of the Central Midwives Board and cannot generally get the best class of work as monthly nurses without a knowledge of gynæcological treatment. Paying probationers are also occasionally received. Probationers must have a fair education. They come for two months' trial, and provide their own uniform. If suitable, they then sign for two years, being paid £8 the first year, £10 the second. The nurses' home is in the same building as the hospital, and the hours of duty are 7 a.m. to 8 p.m., with two hours off duty daily and one and a half hour for meals; a half-day and a whole day once a fortnight, and three weeks' holiday in the year. The sisters and staff-nurses come from general hospitals, with three years' training.

Lectures are given by the resident medical officer on medicine and surgery once a week, the matron lectures after this course, and each ward-sister holds classes for her own probationers, but no examinations are held, as the certificate is given more for practical work than for theory. Each probationer gets theatre work, and a good deal of it. The probationers also work in the out-patient department. Although maternity work is not taught, there are frequent induction cases, Cæsarean sections, and all sorts of obstetric emergencies in the hospital. There are no massage classes, nor is sick-room cookery taught, except incidentally in the wards.

* **Previous articles: May 16, 30; June 13; July 11, 25; Aug. 8, 1914; Jan. 16, 30; Feb. 27; March 13; April 3; May 8 and 22, 1915.**

